# Four Lessons Learned From Career Pivots in Academic Medicine

**DOI:** 10.2196/47641

**Published:** 2023-06-13

**Authors:** Jay-Sheree Allen, Amy Oxentenko

**Affiliations:** 1 Department of Family Medicine Mayo Clinic Rochester, MN United States; 2 Division of Gastroenterology and Hepatology Department of Internal Medicine Mayo Clinic Rochester, MN United States

**Keywords:** women, academic medicine, career, pivot, transition, women in medicine

## Abstract

Women in the fields of medicine and science often consider career pivots to transition out or transition up; in this review, we offer 4 lessons learned to make those pivots maximally successful. These lessons emphasize the need to honor the feeling that it is time to pivot, especially if you develop a strong sense of restlessness indicating you are in a space that no longer serves you; they also emphasize the importance of seeking the guidance of a mentor, sponsor, or coach. Although flexibility is a substantial part of the transition, it is important to have a road map in the form of a career development plan, and it is of utmost importance to complete the transition professionally.

## Introduction

The American Medical Association reports that 1 in 5 physicians plan on leaving their practices within 2 years [1], and in 2021, according to the US Bureau of Labor Statistics, over 47 million Americans voluntarily quit their jobs [2]. The reasons vary but can be summarized by the 5 *R*’s described by Fuller and Kerr [3]: Retirement, Relocation, Reconsideration, Reshuffling, and Reluctance. Simply put, many of these individuals underwent a career pivot. For many women in academic medicine, Respect and Resources (meaning lack thereof) could be added.

For both authors of this viewpoint, career pivots have shaped our recent years and are transforming our futures:

After 4 years in clinical practice preceded by 3 years of residency in Family Medicine, I had to reconsider the current state of my career. Though I enjoyed individual patient care, I was missing the tools needed to have a strong impact on the health outcomes of populations. This led to a major pivot from being an established faculty member to going back to the role of a trainee, this time in Preventive Medicine. The process of this transition has taught me many valuable lessons, but most importantly I have learned to honor the feeling when I know that it is time to pivot, I am able to seek help from the most appropriate source, I have become intentional about creating a career development plan, and I recognize the importance of a professional transition.

Dr Jay-Sheree Allen

I was a career educator. After over 15 years of leadership in education, ranging from fellowship and residency program director roles, to serving on executive education committees locally and nationally, to publishing on curricular elements and training outcomes, I decided to pivot. I loved education leadership passionately, and still do. However, the career path that I was on did not challenge me in ways it once did, and I felt the urge to learn new things and to lead in new ways. So when a new practice leadership role presented itself, I decided to take the leap. Transitioning from a residency program director role to chair of a department to vice dean of practice, I learn something new every day and feel challenged. Leadership skills are translatable across many roles, but that fear of the unknown initially held me back.

Dr Amy Oxentenko

Based on our experiences we offer 4 lessons learned to make those pivots maximally successful.

## Honor the Feeling That It Is Time to Pivot

Some of the most common reasons that contribute to the attrition of women and underrepresented in medicine faculty in academic medicine include a lack of career or professional advancement, salary disparities, and departmental leadership issues, which accounted for up to 166 medical school faculty leaving an institution within a 4-year time frame [4]. Some leave due to the realization that they are not living up to their highest potential, whereas others stay because of the comfort of mastering their current role and loyalty to those who provided the opportunity. Additionally, there are many who have revisited their work-life integration or competing priorities amid the pandemic and felt that a change was necessary. Though we may be able to convince ourselves (temporarily) that a pivot is not necessary, it is not uncommon to develop a strong sense of restlessness when we are no longer in a space that serves us.

It is important to record these feelings and capture both the objective and subjective reasons why we are considering a pivot. As we move through different phases of the transition and continue to grow on our career journey, it is important to be able to look back and remember the reasons for the pivot. This becomes especially noteworthy when we inevitably encounter hard times, and it serves as a reminder that the pivot was still a good decision.

One of the reasons women have such a hard time pivoting is their desire to be loyal, which can lead them to neglect their future, sacrificing their ambitions, and sell themselves short of their true potential. Often, women fear that they may disappoint the individual(s) who sponsored them for their current roles, which often keeps them stuck where they are. In all reality, those who have provided sponsorship in the past are often the ones who are most encouraging of you as you continue to pivot and grow. Similarly, women often focus intently on their current role and address those things that are imminently on their plate rather than taking in the big picture perspective on where they want their career to go. Finally, women are often filled with imposter syndrome as they consider the idea of next roles and often stay in the mastery of their current role to shut down those inner voices that may be telling them “you are not qualified,” when in all reality, they are. A key derailer for leaders is the inability or unwillingness to adapt or pivot. In the book, How Women Rise*,* by Sally Helgesen and Marshall Goldsmith [5], they address the 12 habits that hold women back, and many of them relate to women staying in positions too long.

## Call a Mentor, Sponsor, Coach, or All Three

Career pivots are fraught with high emotions and the potential for career-ending mistakes if they do not play out smoothly. However, they have the potential to lead to significant career growth, so they need to be well thought out to make the most out of the new opportunity. To help navigate some of the potential landmines, it is important to seek the wisdom of individuals with experience. Though mentorship has long been considered the most important type of professional relationship for career development in academic medicine [6], it is not the only service we need when navigating such a significant pivot.

It is important to know the difference between a mentor who “talks with you” about your career, goals, plans, and aspirations versus a coach who “talks at you” as you try to perfect something very specific and a sponsor who “talks about you” when you are not in the room [7].

Networking and fostering relationships can help to build new knowledge, skills, and social capital necessary to transition into a new job role [8]. However, being prepared to share skills and expertise with an up-to-date curriculum vitae, a shorter executive summary, a strong letter of interest (if applicable) and a brief “elevator speech” may increase the likelihood of a successful pivot [9]. The added professional development tool of executive coaching for physicians may have a significant role in supporting productivity, increasing workplace engagement, and transforming the culture of medical practice [10]. Coaching can also provide the input needed of how to approach a current employer about a pivot; provide strategies for an application, interview, and negotiation; and allow us to work through some of the angst that may accompany such pivots. Once in a new role, a coach can also be invaluable in helping us plan out our early wins, navigate unforeseen challenges, and erase the imposter syndrome that may start to develop in a new role.

## Career Development Plan

Though we may be eager to pivot, it is important to have a road map, albeit a flexible one, to guide future career decisions. It is worth spending time to map out the path that you are currently on, listing each of the roles you have held in the past up to the role you currently hold. This can be well visualized on a timeline that also includes significant life-changing events, such as partnership or a new child for whom you are the primary caregiver. Although it would appear that partners and children are a part of our personal lives and not our career development plan, high-achieving young physician-researchers spend an extra 8.5 hours per week on parenting and domestic work [11], which significantly impacts productivity in the professional space.

After taking the time to think about future opportunities, it is worth considering not only your next role but also how that role will position you for any thereafter. We are often wired to think of the next step in the stereotyped career path, yet we do not spend enough time thinking about what skills and opportunities that next role may provide. Perhaps upon reflection, it may become apparent that the next role you are considering really has no roles or opportunities beyond it, so in essence, it is a dead end. For someone at the latter phase of their career, this may be okay, but for someone in early- to midcareer, this may be a career showstopper. When considering your next role, ask yourself if the path you are on leads to a dead end, has any exit ramps for future pivoting, or becomes such a narrow road that can be limiting in terms of opportunity.

In this regard, we need to stop thinking of career paths as a straight line. There are many options to pivot that can bring you on a completely new path altogether, as long as you know to look for those prospects. When considering how to invest in yourself, think about continued development of transferable skills that can be useful in any future role, such as communication skills, emotional intelligence, team building, personnel management, and strategic planning. Taking the focus away from specific roles and titles and instead targeting achievements that you are looking to make will give fulfillment and open doors of opportunity.

## Transition Professionally

By the time we have grappled with the possibility of a pivot, sought out wise counsel with expertise to help guide our decision-making process, and spent the time to develop a career plan, we may be all too eager to close the door on our current situation and step into our new opportunity. It is essential to leave your current role in a professional manner. This means you need to reserve some energy for the transition process to ensure you do not come across as though you have one foot out the door or leave essential duties unfinished. It is worth the time to set your successor up for success if they have been identified. The strong leadership skills you have cultivated, such as interpersonal skills, strategic thinking, and effective communication style, can serve you well as you navigate the transition.

Lastly, do not burn bridges. If you do good work and pivot for good reason, others will understand that and make opportunity. Do not forget—you can and should control the narrative. In your final days, you need to strike a delicate balance between sharing your truthfulness in constructive ways during an exit interview process and ensuring you highlight your appreciation for the many positive things you are taking away from the experience, including having acquired the skills for the candidacy for what lies ahead. Many people leave a role or place of employment thinking they will never come back—but never say never—you want to present yourself in such a way that if you do decide to pivot full circle you will be welcomed with open arms.

## References

[1] Sinsky CA, Brown RL, Stillman MJ, Linzer M (2021). COVID-related stress and work intentions in a sample of US health care workers. Mayo Clin Proc Innov Qual Outcomes.

[2] Penn Rick, Nezamis Eric (2022). Job openings and quits reach record highs in 2021, layoffs and discharges fall to record lows. Monthly Labor Review.

[3] Fuller J, Kerr W (2022). The great resignation didn’t start with the pandemic. Harvard Business Review.

[4] Cropsey KL, Masho SW, Shiang R, Sikka V, Kornstein SG, Hampton CL, Committee on the Status of WomenMinorities‚ Virginia Commonwealth University School of Medicine‚ Medical College of Virginia Campus (2008). Why do faculty leave? Reasons for attrition of women and minority faculty from a medical school: four-year results. J Womens Health (Larchmt).

[5] Helgesen S, Goldsmith M (2018). How Women Rise: Break the 12 Habits Holding You Back from Your Next Raise, Promotion, or Job.

[6] Ayyala MS, Skarupski K, Bodurtha JN, González-Fernández M, Ishii LE, Fivush B, Levine RB (2019). Mentorship is not enough: exploring sponsorship and its role in career advancement in academic medicine. Acad Med.

[7] Gotia R (2022). Why you need a role model, mentor, coach and sponsor. Forbes.

[8] Greer TW, Kirk AF (2022). Overcoming barriers to women's career transitions: a systematic review of social support types and providers. Front Psychol.

[9] Vail EA, Lane-Fall MB (2023). How did they get there? what perspectives from the top tell us about developing women leaders in academic anesthesiology. Anesth Analg.

[10] Kirk VG, Kania-Richmond A, Chaput K (2019). Executive coaching for leadership development: experience of academic physician leaders. Healthc Q.

[11] Jolly S, Griffith KA, DeCastro R, Stewart A, Ubel P, Jagsi R (2014). Gender differences in time spent on parenting and domestic responsibilities by high-achieving young physician-researchers. Ann Intern Med.

